# The Anti-Nociceptive Potential of Tulathromycin against Chemically and Thermally Induced Pain in Mice

**DOI:** 10.3390/pharmaceutics13081247

**Published:** 2021-08-12

**Authors:** Mohamed Elbadawy, Amira Abugomaa, Hussein M. El-Husseiny, Ahmed S. Mandour, Mohamed M. Abdel-Daim, Salama Mostafa Aboelenin, Mohamed Mohamed Soliman, Amany El-Mleeh

**Affiliations:** 1 Department of Pharmacology, Faculty of Veterinary Medicine, Benha University, Moshtohor, Toukh 13736, Elqaliobiya, Egypt; 2 Faculty of Veterinary Medicine, Mansoura University, Mansoura 35516, Dakahliya, Egypt; s193249s@st.go.tuat.ac.jp; 3 Department of Surgery, Anesthesiology, and Radiology, Faculty of Veterinary Medicine, Benha University, Moshtohor, Toukh 13736, Elqaliobiya, Egypt; hussien.alhussieny@fvtm.bu.edu.eg; 4 Department of Veterinary Medicine (Internal Medicine), Faculty of Veterinary Medicine, Suez Canal University, Ismailia 41522, Ismailia, Egypt; dr_mandour@vet.suez.edu.eg; 5 Department of Pharmaceutical Sciences, Pharmacy Program, Batterjee Medical College, P.O. Box 6231, Jeddah 21442, Saudi Arabia; abdeldaim.m@vet.suez.edu.eg; 6 Pharmacology Department, Faculty of Veterinary Medicine, Suez Canal University, Ismailia 41522, Ismailia, Egypt; 7 Biology Department, Turabah University College, Taif University, Taif 21995, Saudi Arabia; s.aboelenin@tu.edu.sa; 8 Clinical Laboratory Sciences Department, Turabah University College, Taif University, Taif 21995, Saudi Arabia; mmsoliman@tu.edu.sa; 9 Department of Pharmacology, Faculty of Veterinary Medicine, Menoufia University, Shebeen Elkoum 32511, Menoufia, Egypt; Amany.ahmed1074@gmail.com

**Keywords:** analgesic, anti-nociceptive, macrolides, tulathromycin, pain

## Abstract

The present study was conducted to evaluate the analgesic potential of the new triamilide macrolide antibiotic, tulathromycin, at 20 and 40 mg/kg of body weight (BW), subcutaneously against acute pain in mice. Acute pain was induced either chemically (using acetic acid-induced writhing and formalin-induced pain tests) or thermally (using hot-plate, and tail-flick tests). In the acetic acid-induced writhing test, tulathromycin induced a dose-dependent and significant decrease in the number of writhes compared with the control group. In the late phase of the formalin test, a significant decline in hind paw licking time compared with the control group was observed. In the hot-plate and tail-flick tests, tulathromycin caused a dose-dependent and significant prolongation of latency of nociceptive response to heat stimuli, compared with the control group. These findings may indicate that tulathromycin possesses significant peripheral and central analgesic potentials that may be valuable in symptomatic relief of pain, in addition to its well-established antibacterial effect.

## 1. Introduction

Pain is an established consequence in almost all illnesses. While uncomfortable, it is a warning of disease or a threat to the body. Pain is the most common reason for physician consultation [1,2] and its control is a substantial event in remedy and comfort of patients.

Nociception (also nocioception or nociperception, from Latin nocere ‘to harm or hurt’) is the response of sensory nervous system to certain stimuli approaching or exceeding harmful intensity (nociceptors). Injuries to the peripheral or central nervous system can elicit a variety of physiological and behavioral responses and usually lead to the report of pain, even in the absence of a noxious stimulus [3]. Several types of stimuli may induce nociceptive pain. Chemical and thermal stimuli are the two major types provoking acute pain through diverse neurobiological pathways. Intense chemical (e.g., chili powder in the eyes, iodine in a cut wound or chemicals released during inflammation as prostaglandins, histamine, bradykinin, etc.), or thermal (heat and cold) stimulation of sensory nerve cells called nociceptors generates nociceptive impulses that stream along a chain of nerve fibers via the spinal cord to the brain [4]. Nociceptive impulses are conducted through either myelinated axons named Aδ-fibers, whose conduction velocity is relatively fast (5~30 ms), or unmyelinated axons called C-fibers with a relatively slow conduction velocity (0.2~2.0 ms). The free nerve endings of Aδ-fibers react well to thermal stimuli, however, those of C-fibers react well to chemical stimuli [5]. The role of nociceptors and ion channels in thermal stimuli- and chemical stimuli-induced acute pain is different [6]. Thus, in analgesic experiments, both approaches should be targeted.

An analgesic is a drug that can relieve pain as a symptom, without affecting its cause [7,8]. It acts in different mechanisms on both peripheral and central nervous systems. They are numerous, involving non-steroidal anti-inflammatory drugs (NSAIDs, like salicylates), and opioid drugs like morphine. The intensity and essence of pain as well as the compatibility with both patient conditions and other co-administered drugs determine the choice of the most suitable analgesic agent [9].

In addition to the well-established, standard analgesics, some other drugs may possess analgesic potentials along with their main pharmacological action. For example, several different antibiotics were proven to decrease pain to different degrees in rats regardless of their type [10,11,12]. This may be beneficial in achieving synergism when these drugs are administered concomitantly with the typical analgesics rendering, sometimes, remedy more effective and reasonable. 

**Figure 1:**
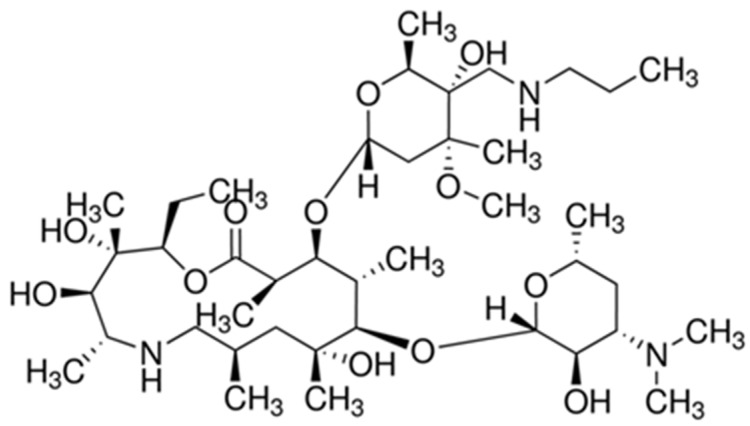
Chemical structure of tulathromycin.

Tulathromycin is a semisynthetic 15-membered-ring triamilide macrolide antibiotic derived from erythromycin [13]. Tulathromycin is a bacteriostatic antibiotic and acts by preventing protein synthesis [14]. It is active against most aerobic and anaerobic Gram-positive and Gram-negative bacteria in bovine and swine including *Mannheimia haemolytica*, *Pasteurella multocida, Histophilus somni, Mycoplasma bovis, Moraxella bovis, Fusobacterium necrophorum, Porphyromonas levii, Helicobacter*, and *Neisseria* species. Tulathromycin has a unique chemical structure (Figure 1), which has three nitrogen/amine functional groups [13], each can be positively charged at the suitable pH with a pKa ranging from 8.6 to 9.6 depending on the basic amino group in the molecule. Additionally, the drug is extremely soluble in hydrophilic environment [15]. Thus, tulathromycin has favorable pharmacokinetic properties including rapid absorption from injection site, extensive tissue distribution, and a long (90 h) plasma elimination half-life in cattle [15,16], thereby providing high and prolonged therapeutic concentrations in lung tissue for 10–15 days after a single administration [16,17]. These favorable properties of tulathromycin allow for a smaller and a single dose to be administered to achieve a high concentration in the target tissue. After a single injection, tulathromycin showed metaphylactic and therapeutic efficacy in bovine and swine respiratory diseases as well as infectious bovine keratoconjunctivitis, and interdigital necrobacillosis in bovine [15,15,18,19,20,21]. Such features make tulathromycin a valuable and potential alternative against violent susceptible bacteria in bovine and swine.

Usually, the prescription for pain- or inflammation-associated infectious diseases involves strong anti-inflammatory analgesic-antipyretic medications together with the main drug of prescription. It will be preferable if that antibacterial agent has, in addition, a pain-killer effect. Although the in vitro inflammatory modulating effects of tulathromycin have been documented by Fisher et al. [22,23,24], there is no data about the in vivo analgesic potential of tulathromycin based on our information. Therefore, the purpose targeted in the current study was to evaluate the in vivo analgesic potential of tulathromycin on the acute pain induced thermally and chemically using various pain models in mice.

## 2. Material and Methods

### 2.1. Chemicals and Equipment

Tulathromycin (100 mg/mL, Draxxin^®^), a ready-to-use sterile aqueous parenteral preparation, was obtained from Zoetis Inc. Kalamazoo, MI 49007, USA and has the molecular formula of C_41_H_79_N_3_O_12_. The formulated high concentration of the drug allows its low-volume dosing. Sterilized distilled water was used to dilute the drug at dose volumes of 0.3 mL equivalent to 20 (small dose) and 40 (large dose) mg/kg BW of mice. Acetic acid and formalin were obtained from El Nasr Pharmaceutical Chemicals Co. (ADWIC), Cairo, Egypt. Ketoprofen sterile injectable solution was obtained from Sanofi-Aventis Co., Cairo, Egypt, under the trade name Profenid^®^ (50 mg ketoprofen/mL). Nalbuphine hydrochloride (Nalufin^®^ ampoule) was obtained as an injectable solution, 20 mg/mL (Amoun Pharmaceutical Co. SAE, Cairo, Egypt). Other chemicals were of analytical grade and locally purchased. 

### 2.2. Experimental Animals

Experiments were conducted on 80 male Swiss albino mice weighing 28 ± 3.2 g and obtained from the Experimental Animal House of the Faculty of Veterinary Medicine, Benha University, Egypt. Mice were housed in polypropylene cages with aspen shavings as a bedding material and were kept in an air-conditioned room at 24 °C and relative humidity of 60% with a 12 h light/dark cycle with free access to standard pellet diet and water. Animals were acclimated for one week before experimentation. Each mouse was used only one time and experimenters were blind to the treatment of mice. All experimental procedures of the current study were conducted according to the guidelines of the Declaration of Helsinki, and approved by the local Ethical Committee of Faculty of Veterinary Medicine, Benha University, Benha, Egypt (approval number: BUFVTM 02-07-20), and all efforts were done to keep the comfort of mice.

### 2.3. Experimental Design

Several mice were checked for the normal reflex to pain by exposing them to thermal stimuli, and only normally responsive mice were selected for the experiments. The experiments were conducted in a parallel design. At random, mice were separated into four main groups, 20 per each. Each group was further split into four subgroups (5 per group) and labeled appropriately. The first and second main groups were used for assessment of the analgesic potential of tulathromycin against chemical stimuli, while the third and fourth ones were specified to check its analgesia against thermal stimuli. 

In the 1st main group, the four subgroups were treated with either a small or large dose of tulathromycin (20 or 40 mg/kg BW, subcutaneously (s.c.), respectively) which are around the tulathromycin dose (28 mg/kg of BW) used previously in mice [25,26], or nalbuphine hydrochloride at 2.2 mg/kg BW, s.c. [27,28] as a standard central analgesic or normal saline as a control, and one hour later, the mice were assigned for the hot-plate test. The s.c. injection of tulathromycin to mice at the two tested doses showed no signs of toxicity except for an abnormal small localized soft lump at the injection site and disappeared within 2 h. 

The 2nd main group was assigned to the tail-flick test where the mice of the four subgroups were treated in the same way as those of the third main group and tested. The volume of all treatments was 0.3 mL, and the performed analgesic tests are described below.

The 3rd main group was used for the acetic acid-induced writhing test; in which, the first and second subgroups were administered a single small and large doses of tulathromycin (20 and 40 mg/kg s.c., respectively), which are around the tulathromycin dose (28 mg/kg of BW) used previously [25,26]; the third subgroup was administered ketoprofen at 5 mg/kg [29,30], as a standard peripheral analgesic; while the fourth subgroup received sterile normal saline s.c. as control. 

The 4th main group was assigned to the formalin test where mice were treated as in the first main group and one hour later, challenged with 20 μL of a 2.5% formalin solution s.c. in the dorsal surface of the right hind paw. 

#### 2.3.1. Hot-Plate Test

The test was performed on the 1st main group following the model described previously [31]. After the animals were treated as mentioned above, they were placed separately into clear Perspex cylinders (30 cm in height and 20 cm in diameter) on the hot-plate (SCILOGEX, Rocky Hill, CT, USA) set at a fixed temperature of 55 °C; the paw licking and/or jumping were defined as responses to the thermal stimulus-induced pain. The time (in seconds) between the contact of mice with the heated plate and responses was reported as the “response latency” and was recorded four times at 1, 2, 3, and 4 h following administration. A “*cutoff*” time of 30 s was applied to minimize tissue damage to mice and the percent of maximal possible effect (MPE%) was calculated. The MPE% is calculated as the percentage of difference between measured response (tulathromycin latency) and the baseline response (latency of the control), divided by the difference between the maximum response (*cutoff* time) and the baseline response (latency of the control) [32,33], and indicated as follows:

$$\mathrm{Percent}\;\mathrm{of}\;\mathrm{MPE}=100\times \left(\frac{\mathrm{Test}\;\mathrm{latency}-\mathrm{control}\;\mathrm{latency}}{Cutoff\;\mathrm{time}-\mathrm{control}\;\mathrm{latency}}\right)$$

#### 2.3.2. Tail-Flick Test

The experiment was performed on mice of the 2nd main group according to the principles as described before [34]. After the treatments mentioned above, the mice were individually confined in a mouse holder with the tail extending out. The end of each mouse’s tail was submerged in the hot water of a thermostatic water bath adjusted at 55 ± 0.5 °C. The time (in seconds) consumed to flick or drag the tail as a response to the painful stimulus was recorded. The reaction times of all mice were reported at 60, 120, 180, and 240 min following administration of vehicle/test drug/standard drug. A “cut-off” period of 10 s was applied to prevent tail injury and MPE% was calculated similarly as in the hot-plate test.

#### 2.3.3. Acetic Acid-Induced Writhing Response Test

The test was performed as described previously [35,36]. Sixty minutes after the various treatments mentioned above, a dose of 0.1% (*v*/*v*) acetic acid (0.1 mL/10 g) was administered intraperitoneally. Five minutes later, mice were placed individually in clear Perspex cylinders for counting the number of writhing responses (a wave of abdominal muscle contractions followed by extension of hind limbs) for 25 min. For the treated subgroup, the mean value was determined and compared with that of the control group. The percentage of analgesia was estimated as follows:

$$\mathrm{Percent}\;\mathrm{of}\;\mathrm{analegesia}=100-\left(\frac{NWt}{NWc}\times 100\right)$$

where:

*NWt* and *NWc* are the number of writhes in the test group and control group, respectively.

#### 2.3.4. Formalin-Induced Paw Licking Test

The formalin test was carried out as described before [37]. One hour after the mice received different treatments as described earlier, 20 µL of 2.5% (*v*/*v*) formalin were injected s.c. into the dorsal surface of the right hind paw of each mouse. Immediately after the injection of formalin, the mice were returned to their cage and observed for 30 min and nociception was evaluated and recorded by stopwatch based on the amount of time spent licking the injected hind paw. The first 5 min post formalin injection is known as the early phase (phase 1) and the period between 20 and 30 min as the late phase (phase 2) [36,38]. The mean value of each treated group was compared with that of the control group and the degree of analgesia in each phase was estimated as follow:

$$\mathrm{Percent}\;\mathrm{of}\;\mathrm{analegesia}=100-\left(\frac{TLt}{TLc}\times 100\right)$$

where:

*TLt* and *TLc* are the total paw licking time in the test group and control group, respectively.

### 2.4. Statistical Analysis

All statistical analysis procedures were carried out by Sigma plot software (Version 14.5; SPSS Inc., Chicago, IL, USA). The obtained data were presented as mean ± SD (*n* = 5 recordings). Statistical significance between control and each treated group was determined using the one-way analysis of variance test (ANOVA) traced by the Bonferroni *t*-test. Normality test, Shapiro–Wilk was performed, and all were successfully passed. Statistical significance was considered when *p* ≤ 0.05. The anti-nociception activity of tulathromycin was standardized as a percentage compared with the corresponding standard in the present study. 

## 3. Results

### 3.1. Analgesic Effect of Tulathromycin against Thermal Stimuli-Induced Pain

**Figure 2:**
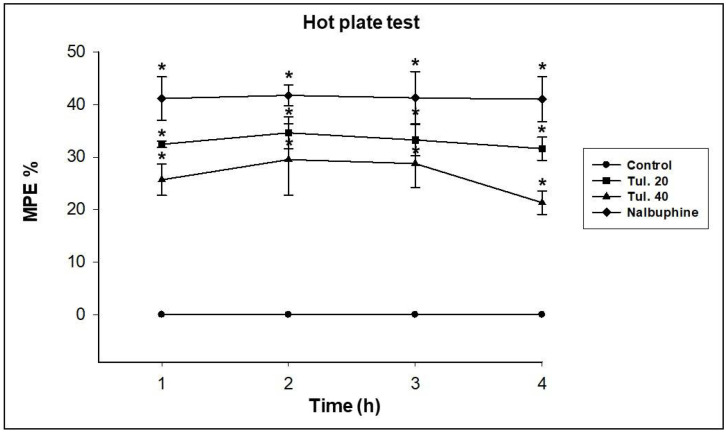
The percentage of maximal possible effect (MPE%) of nalbuphine hydrochloride (2.2 mg/kg BW s.c.) and tulathromycin at 20 (Tul. 20) and 40 (Tul. 40) mg/kg BW s.c. in mice using the hot-plate test. Data are expressed in mean ± SD; *n* = 5. * Significantly different from control, *p* < 0.05.

**Figure 3:**
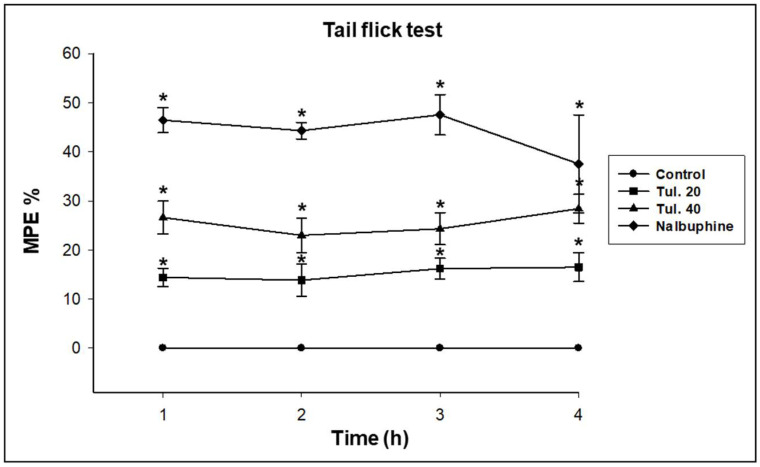
The percentage of maximal possible effect (MPE%) of nalbuphine hydrochloride (2.2 mg/kg BW s.c.) and tulathromycin at 20 (Tul. 20) and 40 (Tul. 40) mg/kg BW s.c. in mice using the tail-flick test. Data are expressed in mean ± SD; *n* = 5. * Significantly different from control, *p* < 0.05.

In both hot-plate test and tail-flick test, administration of tulathromycin at the two tested doses increased significantly (*p* ≤ 0.05) the latency of nociceptive responses to the painful stimuli as well as the MPE% from one to four hours after treatment in a dose-dependent way (Table 1 and Table 2 and Figure 2 and Figure 3, respectively). Further, the standard analgesic drug, nalbuphine hydrochloride at 2.2 mg/kg, s.c., significantly (*p* ≤ 0.05) prolonged the pain tolerance time in the thermal-induced pain experiment in the present study.

### 3.2. Analgesic Effect of Tulathromycin against Chemical Stimuli-Induced Pain

**Figure 4:**
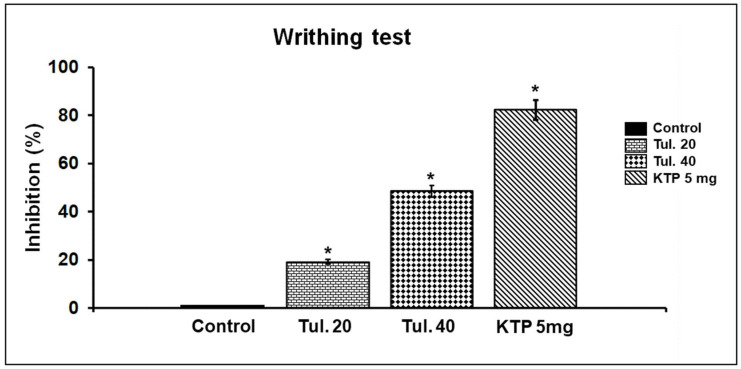
The percentage of inhibition induced by tulathromycin at 20 (Tul. 20) and 40 (Tul. 40) mg/kg BW s.c. and ketoprofen (KTP; 5 mg/kg BW s.c.) against the writhing reflexes triggered by acetic acid (0.1 mL/10 g BW of 0.1% solution (*v*/*v*, i.p.)). Data are expressed in mean ± SD; *n* = 5. * Significantly different from control, *p* < 0.05.

**Figure 5:**
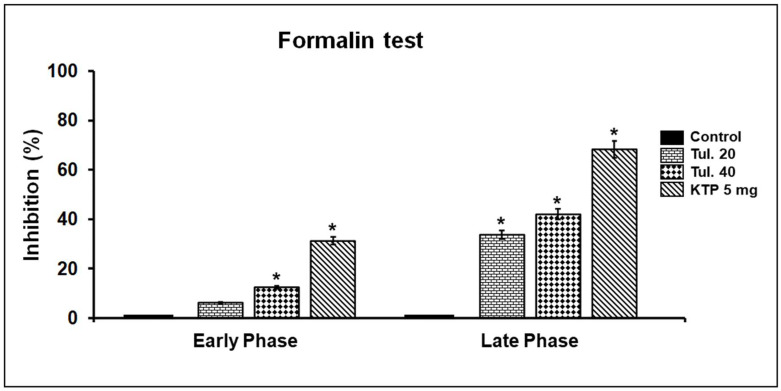
The percentage of inhibition induced by tulathromycin (20 (Tul. 20) and 40 (Tul. 40) mg/kg BW s.c.) and ketoprofen (KTP; 5 mg/kg BW s.c.) against the nociceptive responses triggered by s.c. injection of formalin (20 uL of 2.5%, *v*/*v*) in the dorsum of the hind right paw in mice. Data are expressed in mean ± SD; *n* = 5. * Significantly different from control, *p* < 0.05.

In the acetic acid-induced writhing test, in contrast with vehicle treatment, tulathromycin in a dose-dependent way, significantly (*p* ≤ 0.05) reduced the number of writhing reflexes (Table 3, Figure 4). Additionally, in a similar way, in the formalin test, tulathromycin, in a dose-dependent way, decreased significantly (*p* ≤ 0.05) the time of paw-licking in the second phase only. However, in the first phase of the assay, no significant changes were recorded (Table 4 and Figure 5). The standard analgesic drug, ketoprofen at 5 mg/kg s.c., significantly mitigated the pain symptoms in the chemical-induced pain tests in the current study.

## 4. Discussion

Macrolides, a well-known class of antibiotics, play a substantial role in treating various infections with the advantages of intracellular penetration and accumulation (particularly in phagocytes) [39,40,41], anti-inflammatory [23,42,43], immune-modulating properties [44,45], and improved pharmacokinetics [46]. Such data found a basis for the establishment of novel macrolide derivatives with enhanced tolerance and antimicrobial activity. However, based on our knowledge, there is no published data concerning the analgesic potential of tulathromycin.

Nociception can be controlled or modulated in several ways: removal of the painful stimuli; reducing the sensitivity of nociceptors (by analgesics, antipyretic, and/or local anesthetics); interfering with the nociceptive signaling in sensory nerves (by local anesthetics); inhibiting the conduction of nociceptive impulses in the dorsal spinal cord (by opioids); suppressing the pain perception (by opioids or general anesthetics); and/or modifying emotional reactions to pain in the supraspinal region [47]. 

In the present study, we found that tulathromycin ameliorated both chemically and thermally induced acute pain in mice. The increase of latency response from 1 to 4 h in the hot-plate test as well as the tail-flick test after the s.c. administration of the two tested doses of tulathromycin indicates that tulathromycin produced analgesia against thermal-induced painful stimuli. Additionally, the reduced the number of writhing reflexes in the acetic acid-induced writhing test and the decrease of the time of paw-licking in the formalin test indicates that tulathromycin has the potential to alleviate the chemically induced acute pain, whether of cutaneous (formalin test) or visceral origin (acetic acid test).

In the acetic acid-induced writhing and formalin-induced pain models, pain is indirectly developed by stimulating the affected peripheral tissues to discharge its endogenous inflammatory mediators such as prostaglandins, histamine, bradykinin, serotonin, and substance P, which in turn irritate the nociceptive nerve endings or fibers inducing pain [48]. The developed pain could be diminished by NSAIDs or opioids analgesics as well [49,50]. Interestingly, the decreased writhing and paw licking (at early and late phase) by tulathromycin in the current study was comparable to the used standard analgesic drug, ketoprofen. These data indicate that tulathromycin might reduce the synthesis and discharge of endogenous inflammatory mediators. Additionally, in the formalin test, tulathromycin diminished the paw licking at the early phase (neurogenic phase), which means that the drug acts directly and locally on nociceptors before synthesis of endogenous inflammatory mediators as prostaglandins. Similarly, at the late phase (inflammatory phase), tulathromycin might act by the inhibition of the synthesis and release of inflammatory mediators, the same as ketoprofen. During inflammation, prostaglandin E2 is the most produced and released prostanoid and contributes to fever, pain, and swelling [51]. 

Numerous reports revealed the reduced accumulation of pro-inflammatory mediators by macrolides. Erythromycin, clarithromycin, roxithromycin, azithromycin (a 15-membered-ring macrolide with a nearly similar structure to tulathromycin) were shown to reduce gene expression and production of intercellular adhesion molecule 1 and reduced production of cytokines of IL-6, IL-8, IL-1β, TNFα [52,53,54,55]. Tylvalosin was recently found to possess anti-inflammatory characteristics by inhibiting NF-κB and IL-8 in models of lipopolysaccharide-induced lung inflammation in mice or piglets infected with Porcine Reproductive and Respiratory Syndrome Virus [42]. Additionally, it was shown to inhibit the production of pro-inflammatory mediators, interleukin-8, interleukin 1α, and leukotriene B4, in porcine leukocytes [43]. Since tilmicosin and tylosin also decreased the production of prostaglandin E2 through downregulating COX-2 gene expression [56,57], this antinociception by tulathromycin might be associated with similar effects.

Regardless of their class or type, several different antibiotics were reported to possess dose-dependent antinociceptive properties in rats [11,58] and mice [10,36]. Suaudeau et al. studied the putative analgesic activities of several, randomly selected, and different antibiotics with different doses against acute pain using the hot-plate test in rats [11]. They reported that chloramphenicol or ampicillin administration in a dose range used in human therapy (100 mg/kg), induced comparable analgesia to that of the standard analgesics, salicylate, and ketoprofen [11]. Although chloramphenicol showed a good and long-lasting (>10 h) analgesia, thiamphenicol demonstrated a weak antinociceptive effect. Among the aminoglycosides tested antibiotics, amikacin revealed a potent analgesic effect compared with kanamycin. Penicillins (amoxicillin, ampicillin, and oxacillin) showed different degrees of analgesia with different duration of actions depending on their doses [11]. In another study, streptomycin and neomycin produced central analgesia to inflammatory pain caused by formalin or carrageenan administration in rats by modulating the acid-sensing ionic channel currents in dorsal root ganglion neurons [58,59]. In mice, the centrally administered aminoglycoside (neomycin > gentamicin > kanamycin) induced significant analgesia due to blocking N-type calcium channels and thus lowering the neuronal calcium availability [10]. In rats, injection of formalin into the plantar surface of right hind paw as a pain induction model has dramatically increased the level of ionized calcium-binding adapter molecule 1 as well as the proinflammatory cytokines (IL-6 and IFN-γ), and pain neurotransmitters [60]. Tilmicosin administration was also associated with calcium channel blockade [57,61]. Interestingly, tulathromycin was shown to decrease serum level of ionized calcium [62] which is necessary for nerve conduction and involved in the mechanism of pain [63]. Additionally, tulathromycin was reported to inhibit the nuclear factor-κB signaling [22] that is involved in visceral pain [64]. Further, azithromycin, the structurally similar macrolide to tulathromycin, was reported to inhibit matrix metalloproteinases-9 [65] that is involved in the development of neuropathic pain [66]. Therefore, the obtained central antinociceptive effect by tulathromycin in the present study might be indirectly induced via lowering the serum ionized calcium level or inhibiting the nuclear factor-κB signaling, and/or matrix metalloproteinases-9.

## 5. Conclusions

The present study is the first to report the antinociceptive potential of tulathromycin. The obtained data may indicate that tulathromycin has the potential of being a peripherally and centrally acting analgesic in addition to its basic antibacterial action. Thus, the usage of tulathromycin may provide synergism with the concurrently prescribed standard analgesics. Further, these data might explain the superior efficacy of tulathromycin in respiratory diseases which are often associated with pain and inflammation in animals.

## Figures and Tables

**Table 1:** Effects of tulathromycin (20 and 40 mg/kg BW, s.c.) and nalbuphine hydrochloride (2.2 mg/kg BW, SC) on the latency of nociceptive response induced in the hot-plate test in mice (mean ± SD; *n* = 5).

Group One	Dose, s.c. (mg/kg BW)	Latency of Nociceptive Response (s)			
		After 1 h	After 2 h	After 3 h	After 4 h
Control	NS	6.20 ± 0.63	5.20 ± 0.57	5.64 ± 0.67	5.74 ± 0.25
Nalbuphine HCl	2.2	13.9 ± 0.55 *	18.0 ± 1.23 *	16.8 ± 0.44 *	15.3 ± 0.49 *
Tulathromycin	20	11.3 ± 2.96 *	13.2 ± 1.30 *	14.6 ± 0.60 *	13.4 ± 0.55 *
Tulathromycin	40	14.1 ± 2.8 *	11.1 ± 1.02 *	11.8 ± 0.51 *	10.8 ± 0.84 *

* Significantly different from control (*p* < 0.05; ANOVA followed by Bonferroni *t*-test). NS, normal saline; s.c., subcutaneously; BW, body weight; h, hour.

**Table 2:** Effects of tulathromycin (20 and 40 mg/kg BW, s.c.) and nalbuphine hydrochloride (2.2 mg/kg BW, s.c.) on the latency of nociceptive response induced in the tail-flick test in mice (mean ± SD; *n* = 5).

Group Two	Dose, s.c. (mg/kg BW)	Latency of Nociceptive Response			
		After 1 h	After 2 h	After 3 h	After 4 h
Control	NS	2.30 ± 0.61	2.36 ± 0.42	2.32 ± 0.44	2.22 ± 0.23
Nalbuphine HCl	2.2	7.44 ± 0.44 *	8.34 v 0.42 *	9.30 ± 0.42 *	7.98 ± 0.15 *
Tulathromycin	20	4.01 ± 0.45 *	4.06 ± 0.40 *	4.72 ± 0.83 *	4.50 ± 0.55 *
Tulathromycin	40	5.05 ± 0.89 *	5.88 ± 0.30 *	6.06 ± 0.32 *	5.76 ± 0.27 *

* Significantly different from control (*p* < 0.05; ANOVA followed by Bonferroni *t*-test). NS, normal saline; s.c., subcutaneously; BW, body weight; h, hour.

**Table 3:** Effects of tulathromycin (20 and 40 mg/kg BW, s.c.) and ketoprofen (5 mg/kg BW s.c.) on the acetic acid (10 mL/kg of 0.55% solution, i.p.)-induced writhing reflexes in mice (mean ± SD; *n* = 5).

Group Three	Dose, s.c. (mg/kg BW)	Nociceptive Response	
		Number of Writhes	Inhibition %
Control	NS	65 ± 5.24	0.00
Ketoprofen HCl	5	14.4 ± 3.44 *	77.9 ± 4.62 *
Tulathromycin	20	48.8 ± 3.77 *	24.7 ± 6.0 *
Tulathromycin	40	34.8 ± 4.15 *	46.6 ± 3.13 *

* Significantly different from control (*p* < 0.05; ANOVA followed by Bonferroni *t*-test). NS, normal saline; s.c., subcutaneously; BW, bodyweight.

**Table 4:** Effects of tulathromycin (20 and 40 mg/kg, SC) and ketoprofen (5 mg/kg BW s.c.) on the nociceptive responses triggered by s.c. injection of formalin (20 ul of 2.5%, *v*/*v*) in the dorsum of the hind right paw in mice (mean ± SD; *n* = 5).

Group Four	Dose, s.c. (mg/kg BW)	Total Paw Licking Time (s)			
		Early Phase (0–5 min)	Inhibition %	Late Phase (20–30 min)	Inhibition %
Control	NS	81.4 ± 2.19	0.00	91.6 ± 3.21	0.00
Ketoprofen HCl	5	60.2 ± 3.96 *	26.0 ± 4.79 *	28.2 ± 2.05 *	69.2 ± 2.82 *
Tulathromycin	20	77.4 ± 2.51	4.88 ± 3.21	65.0 ± 3.08 *	29.0 ± 3.92 *
Tulathromycin	40	72.2 ± 4.66 *	11.2 ± 7.30 *	55.0 ± 3.61 *	39.9 ± 4.23 *

* Significantly different from control (*p* < 0.05; ANOVA followed by Bonferroni *t*-test). NS, normal saline; s.c., subcutaneously; BW, bodyweight; min, minutes.

## Data Availability

All relevant data are included in the manuscript.
